# Serum ferritin and primary lung cancer

**DOI:** 10.18632/oncotarget.21518

**Published:** 2017-10-04

**Authors:** Zhongqing Chen, Bo Zhu, Chao Ou, Yuxuan Li

**Affiliations:** ^1^ Department of Clinical Laboratory, Guangxi Medical University Affiliated Tumor Hospital, Nanning 530021, Guangxi Province, P.R. China; ^2^ Experimental Research Department, Guangxi Medical University Affiliated Tumor Hospital, Nanning 530021, Guangxi Province, P.R. China; ^3^ Department of Hepatobiliary Surgery, Guangxi Medical University Affiliated Tumor Hospital, Nanning 530021, Guangxi Province, P.R. China

**Keywords:** primary lung cancer, Serum ferritin, Hemoglobin, Transferrin, Menopause

## Abstract

Existing research yields conflicting results regarding the relation between iron deficiency and high serum ferritin (SF) levels in primary lung cancer patients. We investigated the concentrations of SF, hemoglobin (Hb) and transferrin (TRF) in 569 male primary lung cancer patients and 252 female primary lung cancer patients. We grouped the subjects according to gender, smoking status, menopausal status, pathological type, stage, and TNM stage. The levels of SF and TRF were correlated with T stage in male patients (*p<0.01*). The levels of SF and TRF were correlated with menopausal status in female patients (*p<0.01*). Hb was correlated with smoking status, pathological type, stage, and TNM stages in male patients(*p<0.01*), but in female patients, Hb was not correlated with these grouping factors(*p>0.05*). The levels of SF may be regulated by different mechanisms and may be of different physiological significance in different populations.

## INTRODUCTION

Recent work has shown that iron has a role in the tumor microenvironment and in metastasis [1]. Cancer cells exhibit an enhanced dependence on iron relative to their normal counterparts, a phenomenon called iron addiction [2, 3]. A higher intake of heme iron has shown a tendency toward a positive relation with cancer risk [4]. The World Health Organization recommended that serum ferritin (SF) concentrations was the best indicator of iron deficiency [5]. So SF has aroused our concern. SF is highly expressed in tumor tissues and serum of patients with non-small cell lung cancer [6]. High expression of SF in non-small cell lung cancer may be also the result of inflammation and oxidative stress [6]. Infection or inflammation generate anemia and profound changes in iron metabolism, and SF increases with infections [7, 8]. Microbes have an essential need for iron, which is required for many microbial metabolic processes and for microbial pathogenicity [9]. SF levels could be used to differentiate between fever of unknown origin caused by infectious and noninfectious diseases [10, 11]. These changes are important confounders to consider in assessments of SF status.

However, studies have shown that iron deficiency in lung cancer patients is as high as 50.7% [12]. One study found that the high SF levels are not equal to high iron content of SF. In this study, the iron content of SF was below the normal reference values in 90% Hemodialysis patients [13], which is also found in non-small cell lung cancer patients [6]. There might be the presence of oligoelements other than iron inside the SF core [13]. A dietary survey of a region with a high incidence of lung cancer in Southwest China found that cadmium and titanium were significantly higher in the lung cancer group than in the control group [14]. Based on these studies, we speculated that SF containing oligoelements stimulated tissues to produce tumors. Tumor tissue also stimulated the body to produce more SF, and SF stored oligoelements for tumor cell consumption.

Existing research yields conflicting results regarding the relation between iron deficiency and high SF levels in primary lung cancer patients. The contradiction between iron deficiency and high SF levels in primary lung cancer patients is more prominent in advanced diseases, deterioration, and solid tumors, such as lung tumors [6, 12]. There is not much clinical research on the relation between SF and primary lung cancer. We will investigate the relation between SF and pathological types, tumor-nodes-metastasis (TNM) stage, smoking status, menopausal status. Besides, hemoglobin (Hb) and transferrin (TRF). We are going to discuss by gender, since the SF levels and Hb levels in women are different from in men.

## RESULTS

### Patients' characteristics

A total of 830 patients were initially diagnosed as primary lung cancer. 3 female smokers and 6 male former smokers were excluded due to they were uncommon cases. 821 patients with primary lung cancer were included in the study. Table 1 summarizes patients’ characteristics of age, smoking status, menopausal status, pathological type, stage, TNM stage. At the time of diagnosis, the male patients ranged from 28 to 86 years of age with an average age of 58.9 years, and female patients aged from 25 to 80 years of age with an average age of 54.7 years. In male patients, the proportion of smokers was greater than non-smokers. Adenocarcinoma was the most common pathological type of lung cancer in both male patients and female patients. The proportion of advanced lung cancer was higher than that of early lung cancer at initial diagnosis.

**Table 1:** Patients' characteristics

Parameters	Men		Women	
	N	%	N	%
Number of patients	569		252	
Smoking status				
Smoker	414	72.8	0	0.0
Never	155	27.2	252	100.0
Menopause				
Yes			180	71.4
No			72	28.6
Pathologic type				
Adenocarcinoma	301	52.9	197	78.2
Squamous cell carcinoma	171	30.1	25	9.9
Adeno-squamous carcinoma	18	3.2	8	3.2
Small cell carcinoma	53	9.3	8	3.2
Not classifiable/Others	26	4.6	14	5.6
Stage				
I	96	16.9	64	25.4
II	46	8.1	17	6.7
III	194	34.1	60	23.8
IV	182	32.0	97	38.5
Not classifiable	51	9.0	14	5.6
TNM				
T1	76	13.4	62	24.6
T2	223	39.2	106	42.1
T3	123	21.6	37	14.7
T4	87	15.3	25	9.9
Tx (Not classifiable)	60	10.5	22	8.7
N0	131	23.0	76	30.2
N1	54	9.5	21	8.3
N2	203	35.7	88	34.9
N3	121	21.3	45	17.9
Nx (Not classifiable)	60	10.5	22	8.7
M0	337	59.2	141	56.0
M1	181	31.8	97	38.5
Mx (Not classifiable)	51	9.0	14	5.6

### The characteristics of SF, Hb, TRF in male primary lung cancer patients

The characteristics among SF, Hb, TRF and smoking status, pathological types, stage, TNM stage in male primary lung cancer patients are shown in Table 2. After chi-square test, there was no difference in the constituent ratio of smoking patients and non-smoking patients in different groups (*p>0.05*) (Supplementary Table 2). Smoking bias had been excluded in groups of pathological type, stage and TNM stage.

**Table 2:** X ± s, above/below the normal rang of SF, Hb, TRF in male primary lung cancer patients

Parameters	Serum ferritin			Hemoglobin			Transferrin		
	x ± s (ug/l)	above (n)	above(%)	x ± s (g/l)	below (n)	below(%)	x ±s (g/l)	below (n)	below(%)
Smoking status	*P=0.037*			*P=0.003*			*P=0.718*		
Smoker	378.7±159.1	354	85.5	130.9±18.2	108	26.1	2.2±0.6	226	54.6
Never	347.7±153.6	122	78.7	135.5±15.1	20	12.9	2.2±0.6	83	53.5
Pathologic type^*^	*P=0.486*			*P=0.005*			*P=0.417*		
Adenocarcinoma	365.8±165.3	247	82.1	134.7±17.0	57	18.9	2.3±0.6	156	51.8
Squamous cell carcinoma	374.9±148.0	147	86.0	128.9±18.6	48	28.1	2.2±0.5	94	55.0
Adeno-squamous carcinoma	319.9±157.4	13	72.2	125.4±19.0	7	38.9	2.2±0.4	11	61.1
Small cell carcinoma	394.0±148.4	48	90.6	131.4±15.7	10	18.9	2.3±0.6	29	54.7
Not classifiable/Others	376.8±157.8	21	80.8	130.8±14.1	6	23.1	2.1±0.5	19	73.1
Stage^*^	*P=0.055*			*P=0.000*			*P=0.154*		
I	334.3±167.1	72	75.0	137.7±13.8	9	9.4	2.3±0.5	41	42.7
II	393.3±164.6	39	84.8	136.3±13.8	8	17.4	2.3±0.7	24	52.2
III	366.2±158.6	162	83.5	128.3±19.2	61	31.4	2.2±0.5	110	56.7
IV	390.5±148.2	161	88.5	132.2±18.1	41	22.5	2.2±0.6	106	58.2
Not classifiable	359.9±159.1	42	82.4	132.7±14.8	9	17.6	2.2±0.5	28	54.9
T^*^	*P=0.000*			*P=0.004*			*P=0.005*		
T1	313.5±167.7	54	71.1	138.1±13.5	8	10.5	2.4±0.6	36	47.4
T2	358.6±163.7	181	81.2	132.3±17.0	50	22.4	2.3±0.6	114	51.1
T3	421.7±135.3	115	93.5	128.2±19.7	41	33.3	2.1±0.5	80	65.0
T4	383.7±143.5	77	88.5	131.9±19.3	18	20.7	2.3±0.6	44	50.6
Tx (Not classifiable)	360.2±160.1	49	81.7	132.5±14.5	11	18.3	2.2±0.5	35	58.3
N^*^	*P=0.133*			*P=0.004*			*P=0.049*		
N0	351.2±164.7	105	80.2	136.5±14.7	15	11.5	2.3±0.5	64	48.9
N1	378.0±154.0	47	87.0	134.7±16.1	11	20.4	2.3±0.7	28	51.9
N2	365.1±151.5	170	83.7	130.3±18.5	50	24.6	2.3±0.5	104	51.2
N3	401.0±160.5	105	86.8	129.1±19.6	41	33.9	2.1±0.5	78	64.5
Nx (Not classifiable)	360.2±160.1	49	81.7	132.5±14.5	11	18.3	2.2±0.5	35	58.3
M^*^	*P=0.140*			*P=0.967*			*P=0.752*		
M0	361.5±162.7	274	81.3	132.1±17.6	78	23.1	2.2±0.5	175	51.9
M1	389.5±148.0	160	88.4	132.2±18.1	41	22.7	2.2±0.6	106	58.6
Mx (Not classifiable)	359.9±159.1	42	82.4	132.7±14.8	9	17.6	2.2±0.5	28	54.9

^*^ *P*>0.05. χ^2^ test in the composition of smoking patients and non-smoking patients.

Hb levels were significantly different in smoking status, pathologic types, stage, T stage and N stage (*p <0.01*). The prevalence of anemia among smokers was twice that of non-smokers. Squamous cell carcinoma and adeno-squamous carcinoma were two pathologic types with high prevalence of anemia in male primary lung cancer patients. The higher the stage, the higher the prevalence of anemia. But the prevalence of anemia in stage III was higher than in stage IV. This phenomenon was the same in T3 stage and N3 stage, and this should be noted. Metastasis had no effect on anemia (*p> 0.05*). The mean SF levels in all groups exceeded the normal range (15 ug/l-200 ug/l). SF levels and TRF levels were significantly different in T stage (*p <0.01*).

### The characteristics of SF, Hb, TRF in female primary lung cancer patients

The characteristics among SF, Hb, TRF and menopausal status, pathological types, stage, TNM stage in female primary lung cancer patients are shown in Table 3. After chi-square test, there was no difference in the constituent ratio of postmenopausal patients and premenopausal patients(*p>0.05*) (Supplementary Table 1). Menopausal bias had been excluded in groups of pathological types, stage and TNM stage.

**Table 3:** X ± s, above/below the normal rang of SF, Hb, TRF in female primary lung cancer patients

Parameters	Serum ferritin			Hemoglobin			Transferrin		
	x ± s (ug/l)	above (n)	above (%)	x ± s (g/l)	below (n)	below(%)	x ±s (g/l)	below (n)	below (%)
Menopause	*P=0.000*			*P=0.112*			*P=0.008*		
Yes	259.7±132.1	144	80.0	123.9±14.6	24	13.3	2.3±0.5	72	40.0
No	121.8±123.3	19	26.4	120.7±14.1	12	16.7	2.6±0.7	22	30.6
Pathologic type^*^	*P=0.645*			*P=0.424*			*P=0.149*		
Adenocarcinoma	224.7±143.9	132	67.0	123.5±14.8	27	13.7	2.5±0.6	69	35.0
Squamous cell carcinoma	197.9±146.6	15	60.0	121.8±13.0	4	16.0	2.2±0.6	11	44.0
Adeno-squamous carcinoma	184.0±155.8	3	37.5	122.0±9.4	1	12.5	2.2±0.7	5	62.5
Small cell carcinoma	174.6±113.9	3	37.5	113.5±16.3	3	37.5	2.3±0.4	3	37.5
Not classifiable/others	244.6±149.8	10	71.4	123.5±14.5	1	7.1	2.2±0.5	6	42.9
Stage^*^	*P=0.546*			*P=0.344*			*P=0.348*		
I	198.7±136.4	39	60.9	125.1±12.8	8	12.5	2.5±0.6	19	29.7
II	216.5±120.2	12	70.6	118.2±17.5	4	23.5	2.3±0.4	6	35.3
III	244.0±156.3	43	71.7	124.0±13.0	8	13.3	2.3±0.5	24	40.0
IV	220.2±139.5	60	61.9	122.3±14.9	12	12.4	2.4±0.6	37	38.1
Not classifiable	222.1±176.5	9	64.3	119.4±20.1	4	28.6	2.3±0.6	8	57.1
T^*^	*P=0.153*			*P=0.086*			*P=0.058*		
T1	192.3±119.5	42	67.7	126.6±13.0	8	12.9	2.6±0.5	14	22.6
T2	225.9±148.5	65	61.3	123.0±12.8	11	10.4	2.4±0.6	37	34.9
T3	266.4±158.6	28	75.7	118.9±17.4	8	21.6	2.3±0.6	18	48.6
T4	205.4±135.9	14	56.0	119.7±16.3	5	20.0	2.3±0.7	14	56.0
Tx (Not classifiable)	211.2±155.5	14	63.6	123.4±17.6	4	18.2	2.4±0.7	11	50.0
N^*^	*P=0.101*			*P=0.787*			*P=0.038*		
N0	197.0±135.7	45	59.2	124.2±14.2	10	13.2	2.5±0.6	22	28.9
N1	186.9±111.3	12	57.1	123.6±9.8	1	4.8	2.6±0.7	6	28.6
N2	228.1±140.3	60	68.2	122.8±15.6	14	15.9	2.3±0.5	35	39.8
N3	264.3±163.2	32	71.1	120.7±13.3	7	15.6	2.2±0.6	20	44.4
Nx (Not classifiable)	211.2±155.5	14	63.6	123.4±17.6	4	18.2	2.4±0.7	11	50.0
M^*^	*P=0.999*			*P=0.488*			*P=0.661*		
M0	220.1±144.1	94	66.7	123.8±13.6	20	14.2	2.4±0.6	49	34.8
M1	220.2±139.5	60	61.9	122.3±14.9	12	12.4	2.4±0.6	37	38.1
Mx (Not classifiable)	222.1±176.5	9	64.3	119.4±20.1	4	28.6	2.3±0.6	8	57.1

^*^ *P*>0.05. χ^2^ test in the composition of postmenopausal patients and premenopausal patients.

Menopausal status, pathological type, stage, TNM stage has no significant effect on anemia. Hb levels did not make any difference in different groups (*p>0.05*). SF and TRF levels were not statistically different in the pathological types, stage and TNM stage in female lung cancer patients (*p>0.05*). SF levels in postmenopausal patients were significantly higher than those in premenopausal patients (*p<0.01*), while TRF levels were just the opposite (*p<0.01*).

### Spearman correlations between Hb, SF and TRF in different groups

Spearman correlation coefficients between Hb, SF and TRF in male patients, postmenopausal patients, premenopausal patients are shown in Table 4. In male patients, TRF levels were positively correlated with Hb (*p<0.01*), and negatively correlated with SF (*p<0.01*). In postmenopausal patients, TRF levels were positively correlated with Hb (*p<0.01*). TRF levels were negatively correlated with SF in premenopausal patients (*p<0.01*). SF levels were not correlated with Hb in all three groups (*p>0.05*).

**Table 4:** *Spearman* correlation between Hb, SF and TRF values in different groups

Parameters		SF	Hb	TRF
Men	SF	1.000		
	Hb	-0.067	1.000	
	TRF	-0.257^**^	0.366^**^	1.000
Postmenopause	SF	1.000		
	Hb	0.042	1.000	
	TRF	-0.130	0.270^**^	1.000
Premenopause	SF	1.000		
	Hb	0.042	1.000	
	TRF	-0.390^**^	0.174	1.000

^**^ *P*<0.01.

SF, Serum ferritin; Hb, Hemoglobin; TRF, Transferrin.

## DISCUSSION

In both animals and humans, primary neoplasms develop at body sites of excessive iron deposits [15]. There are no significant differences in expression of SF between different pathologic types (squamous cell carcinoma or adenocarcinoma) of lung cancer [6]. Consistent with our findings both in men (*p>0.05*) and women (*p>0.05*), SF levels were not statistically different in different pathological types. Strong SF expression was observed in 62% tumor samples, while it is relatively rare in tumor stroma or normal lung tissue samples (12%, 9%) [6]. which revealed that SF levels were significantly different in T stage (*p<0.01*). There exists a clinically relevant relation between SF concentration and the prognosis of survival in lung cancer patients [15–17], even after adjustment for performance status, age, sex, TNM stage, and histological tumor type [16]. SF may be of no benefit in distinguishing the pathological types, stage, N stage, M stage of primary lung cancer (*p>0.05*). There is further evidence that iron content of SF showed positive correlation with iron metabolic parameters and survival [6]. The routine uses of SF, free iron, iron content of SF should be considered in the evaluation and follow-up, which will better assess the status of cancer patients.

In male primary lung cancer patients, anemia prevalence is the highest in stage III, T3, N3. In addition, T3 has the highest SF average levels, the highest proportion of SF above the normal range, the lowest TRF average and the highest proportion of TRF below the normal range. Squamous cell carcinoma and adeno-squamous cell carcinoma have higher rates of anemia. The phenomenon was not found in women. We note that the anemia rate in women with small cell lung cancer is obviously higher than that of other pathological types (37.5%). But because of the small number of samples of squamous cell carcinoma, adeno-squamous cell carcinoma and small cell carcinoma, the correlation between pathological type and anemia in female primary lung cancer patients cannot be determined for the time being. In some studies, the anemia rate in lung cancer patients is 57.6% [6], 57.9% [18], higher than our study. Their subjects were in or after anti-tumor therapy. Chemotherapy causes myelosuppression [19] and surgery leads to blood loss, which increases anemia rates. Pretreatment of anemia can improve the overall survival rate of lung cancer and control disease progression [20].

The large scale of epidemic results showed the significantly negative relations between SF levels and total testosterone, free testosterone, estradiol and sex hormone-binding protein in men, there was borderline negative relation between SF levels and estradiol in men [21]. However, there is no such big data for women. Menopause may be the common link that resulted in higher SF in women [22, 23]. Postmenopausal women who take estrogen have lower levels of SF than those who do not take estrogen [24]. High estrogen levels increase TRF levels [25]. Premenopausal women have higher carbohydrate-deficient TRF levels than postmenopausal women [26, 27], which was similar to our findings. TRF levels in premenopausal patients were significantly higher than those in postmenopausal patients (*p<0.01*). SF levels in men were higher than those in women, and the levels of SF in postmenopausal women were higher than those in premenopausal women, so blood loss monthly was also an important factor in maintaining low and normal SF levels. Blood loss monthly did not decrease Hb levels (*p>0.05*). TRF levels were negatively correlated with SF in premenopausal patients (*p<0.01*), TRF levels were positively correlated with Hb in postmenopausal patients (*p<0.01*). While in male patients, TRF levels were both positively correlated with Hb (*p<0.01*) and negatively correlated with SF (*p<0.01*). We were not clear of the reason. We speculated that SF, TRF, Hb levels were regulated by different mechanisms and were of different physiological significance in men, postmenopausal women, premenopausal women. Considering tumor patients, it may be more complicated.

Our results showed SF levels in smokers with primary lung cancer were not significantly higher than non-smokers with primary lung cancer (*p=0.037*). There is recent evidence that the non-smoking and smoking healthy men exhibited no difference in SF levels [28]. Higher SF levels are associated with a higher Body Mass Index [21, 28]. However, another study shows that smoking has an effect on SF. The reductants present in cigarette smoke can readily mobilize iron from SF, leading to lipid peroxidation and cell damage [29]. More research and data are needed to discover the relation between smoking and SF. But smoking had an obvious effect on anemia. Smokers had a lower Hb level than non-smokers, and anemia was twice as high as non-smokers.

SF has emerged as an excellent and promising protein-based nanocage thanks to its unique architecture, surface properties and high biocompatibility. SF nanocages may ensure a proper drug delivery and release [30–32], such as cisplatin, carboplatin, gefitinib, doxorubicin [33–36]. Based on the results of this study, the next step was to investigate the efficacy of SF encapsulated chemotherapeutic agents in the treatment of primary lung cancer, with a focus on the effectiveness of the treatment and the diversity in the different populations.

SF has the ability to store iron [5], participate in inflammation and infection [7–10], participate in cancer and other functions [1, 2, 15]. SF may represent different functions at different stages of different populations. This may be related to the iron content of SF. Although many of the functions of SF have been discovered and exploited, more advanced and sophisticated detection methods are needed to distinguish between different functions of SF, more prospective work is needed to confirm SF's more functions in disease.

## MATERIALS AND METHODS

### Patients

This study was approved by the ethics committee of the Guangxi Medical University Affiliated Tumor Hospital with an Ethics approval number of KS2016(36). When the patient suspected he had lung cancer, they were first admitted to the thoracic tumor surgery department. We systematically reviewed all patients who had been hospitalized in the thoracic oncology department from October 2014 to April 2017. Cases with other malignancies and/or no pathology and/or no imaging were excluded. The clinical and pathological diagnosis of primary lung cancer was based on the seventh edition of the TNM classification for lung cancer [9–11]. All cases had not been treated with anti-tumor therapy, such as surgery, chemotherapy, radiotherapy, biological therapy, endocrine therapy, Chinese medicine treatment, hyperthermia and radiofrequency ablation therapy. Complete and detailed data were available for all cases.

### Imaging examination

Patients were scanned by 5.0 mm slice thickness from the thoracic cavity to the septum using Siemens 64 row CT machine. All scans were read and diagnosed by two or more senior imaging doctors independently.

### Pathological examination

All primary lung cancer patients underwent surgical biopsy, biopsy under ultrasound or conventional bronchoscopy biopsy. Diagnostic results were given by at least two senior pathologists independently.

### Laboratory assays

Each patient agreed to undergo fasting blood the next morning after admission, which is a routine medical practice. The results of the blood test were obtained on that day. SF and TRF were assayed using Siemens ADVIA 2400. Hb was assayed using Mindray BC-6900. All assays were performed according to instrument and reagent specifications, with strict whole process quality control monitoring of the results. The results were accurate. The normal ranges of men's SF, Hb, and TRF were 15 ug/l-200 ug/l, 120g/l-160g/l, 2.2g/l-4g/l; The normal ranges of women's SF, Hb, and TRF were 12 ug/l-150 ug/l, 110g/l-150g/l, 2.2g/l-4g/l.

### Statistical analyses

IBM SPSS21.0 software was applied for statistical analysis. The levels adopted for significance were *p*>0.05 and *p<0.01* (two-tailed). Numerical variables were represented as mean ± standard deviation (*x±s*). Variance analysis was used to compare the deference for multiple groups and non-paired *t*-test was used to analyze the two groups after homogeneity of variance test. χ^2^-test was used to compare the constituent ratio of two groups. Correlations between parameters were done by *Spearman* correlation.

## SUPPLEMENTARY MATERIALS TABLES



## Abbreviations

SF: serum ferritin
TNM: tumor-nodes-metastasis
Hb: hemoglobin
TRF: transferrin
