# Supplementary material for: Serum ferritin and primary lung cancer
Source: Oncotarget. 2017 Oct 4;8(54):92643–51. doi: 10.18632/oncotarget.21518 (PMC5696211; doi:10.18632/oncotarget.21518)
Supplement: Supplementary file 1 [file oncotarget-08-92643-s001.pdf]

Serum ferritin and primary lung cancer

SUPPLEMENTARY MATERIALS

Supplementary Table 1: Menopause: The number of postmenopausal patients and premenopausal patients in different subgroups (χ^2)

Parameters		Menopause		total
		Yes	No	
T *P=0.125*	T1	49	13	62
	T2	75	31	106
	T3	29	8	37
	T4	14	11	25
	Tx (Not classifiable)	13	9	22
	total	180	72	252
N *P=0.074*	N0	55	21	76
	N1	12	9	21
	N2	71	17	88
	N3	29	16	45
	Nx (Not classifiable)	13	9	22
	total	180	72	252
M *P=0.233*	M0	106	35	141
	M1	66	31	97
	Mx (Not classifiable)	8	6	14
	total	180	72	252
Staging *P=0.360*	I	45	19	64
	II	13	4	17
	III	48	12	60
	IV	66	31	97
	Not classifiable	8	6	14
	total	180	72	252
Pathological Types *P=0,139*	Adenocarcinoma	145	52	197
	Squamous cell carcinoma	19	6	25
	Adenosquamous carcinoma	3	5	8
	Small cell carcinoma	5	3	8
	Not classifiable	8	6	14
	total	180	72	252

Supplementary Table 2: Smoking: The number of smoking patients and non-smoking patients in different subgroups (χ^2)

Parameters		Smoking		total
		No	Yes	
T *P=0.461*	T1	24	52	76
	T2	61	162	223
	T3	27	96	123
	T4	23	64	87
	Tx (Not classifiable)	20	40	60
	total	155	414	569
N *P=0.072*	N0	39	92	131
	N1	20	34	54
	N2	42	161	203
	N3	34	87	121
	Nx (Not classifiable)	20	40	60
	total	155	414	569
M *P=0.241*	M0	83	254	337
	M1	56	125	181
	Mx (Not classifiable)	16	35	51
	total	155	414	569
Staging *P=0.078*	I	32	64	96
	II	12	34	46
	III	39	155	194
	IV	56	126	182
	Not classifiable	16	35	51
	total	155	414	569
Pathological types *P=0.063*	Adenocarcinoma	93	208	301
	Squamous cell carcinoma	41	130	171
	Adenosquamous carcinoma	5	13	18
	Small cell carcinoma	7	46	53
	Not classifiable	9	17	26
	total	155	414	569
